# HIV Knowledge, Self-Perception of HIV Risk, and Sexual Risk Behaviors among Male Tajik Labor Migrants who inject Drugs in Moscow

**DOI:** 10.21203/rs.3.rs-2972274/v1

**Published:** 2023-06-02

**Authors:** Casey Morgan Luc, Judith Levy, Mahbat Bakhromov, Jonbek Jonbekov, Mary E. Mackesy-Amiti

**Affiliations:** University of Illinois at Chicago; University of Illinois at Chicago; PRISMA Research Center; PRISMA Research Center; University of Illinois at Chicago

**Keywords:** HIV Risk Self-Perception, HIV Knowledge, Sexual risk behavior, injection drug use, Tajik migrant worker

## Abstract

**Background:**

The interplay of HIV knowledge and self-perception of risk for HIV among people who inject drugs is complex and understudied, especially among temporary migrant workers (MWID) who inject drugs while in a host country. In Russia, Tajik migrants make up the largest proportion of Moscow’s foreign labor. Yet, HIV knowledge and self-perceived risk in association with sexual risk behavior among Tajik MWID in Moscow remains unknown.

**Objective:**

This research examines knowledge about HIV transmission, self-perception of HIV risk, and key psychosocial factors that possibly contribute to sexual risk behaviors among male Tajik MWIDs living in Moscow.

**Methods:**

Structured interviews were conducted with 420 male Tajik MWIDs. Modified Poisson regression models investigated possible associations between major risk factors and HIV sexual risk behavior.

**Results:**

Of the 420 MWIDs, 255 men (61%) reported sexual activity in the last 30 days. Level of HIV knowledge was not associated in either direction with condom use or risky sexual partnering, as measured by sex with multiple partners or female sex workers. Higher self-perceived HIV risk predicted less risky sexual partnering, but not condom use. Depression and police-enacted societal stigma were positively associated with risky sexual partnering, while loneliness and depression were associated with condomless sex.

**Conclusions:**

HIV prevention programing for male Tajik MWIDs must go beyond solely educating about factors associated with HIV transmission to include increased awareness of personal risk based on engaging in these behaviors. Additionally, psychological services to counter loneliness, depression, and societal stigma through police harassment are needed.

## Introduction

Research has shown that labor migration from low to high human immunodeficiency virus (HIV) prevalence countries is associated with increased risk of HIV infection for migrant workers^1,2^ This risk is further heightened among migrants who inject drugs (MWID) while in the host country. ^3^ In Russia, a country with high HIV prevalence,^4^ HIV risk for MWID is exacerbated by disparities in the availability and provision of HIV prevention and treatment services for labor migrants.^3,5–7^ In addition, MWIDs are impacted negatively by Russian policing practices that focus on enforcing petty criminal justice penalties for migrants in general and toward MWID in particular through physical harassment and syringe confiscation to discourage drug use. Such practices hinder harm reduction efforts and create barriers for MWID in accessing services for the prevention, treatment and care of HIV.^8,9^ Meanwhile, poor working and living conditions, loneliness due to separation from family and friends at home, societal stigma, and the general emotional stress common to temporary migrant laborers in diaspora are believed to increase their vulnerability to HIV through engaging in risky sexual behavior.^5,6^

This research present findings on HIV knowledge, perception of HIV risk, psychosocial factors, and the prevalence and correlates of HIV sexual risk behavior among male Tajik MWID who are temporarily living and working in Moscow. While in Moscow, MWID experience the “double jeopardy” of social marginalization due to their dual status as a migrant and injection drug user, both of which are thought to increase sexual risk for HIV.^3,10^ Meanwhile, HIV knowledge and HIV risk perception have been found to influence sexual risk behavior among those who inject drugs,^11,12^ but the interplay of these two factors on sexual risk behavior is complex and little understood. For example, while a cross-sectional study partly comprised of people who inject drugs (PWID) identified a “relatively good” level of comprehensive knowledge of HIV and acquired immunodeficiency syndrome (AIDS), the prevalence of HIV risk perception was low while sexual risk behavior was high.^13^ These findings suggest that engaging in risky sexual behavior may be best explained by self-perception of being at HIV risk rather than degree of HIV knowledge alone. Yet this premise has yet to be investigated and reported in the scientific literature.

To help address this omission, this study examines the possible effects of HIV knowledge and self-perception of risk for HIV on sexual behavior among Tajik MWID while in Moscow. Also, personal and psychosocial factors appear to influence sexual risk behavior among PWID while possibly playing a role in promoting sexual risk behavior among MWID.^11–13^ In addition, aggressive policing tactics and harassment that reflect government-enacted stigma toward migrants and MWID may form a “driving force” that increases MWID engagement in HIV sexual risk behavior.^14^ Consequently, in addition to examining HIV knowledge and self-perceived risk, the analysis also investigates the possible effects of demographic and key psychosocial factors (depression, loneliness, and societal stigma toward migrants as manifested through police harassment) on sexual risk behavior. Identifying the key factors that influence sexual risk behaviors among MWID as an understudied population may help to inform prevention strategies for successfully addressing their sexual risk while adding to a better scientific understanding of the relationship between risky sexual behavior and HIV knowledge and HIV risk perception.

## Methods

The research design and procedures of this study were reviewed and approved by the institutional review boards of the University of Illinois Chicago, PRISMA Research Center, and the Moscow Nongovernment Organization, “Bridge to the Future.” The analysis is based on data collected at baseline for a clinical trial assessing the efficacy of the MASLIHAT peer-education model in reducing HIV risk behavior among Tajik MWID while living in Moscow.

### Recruitment:

From October 2021 to April 2022, 140 male Tajik migrant workers who inject drugs were recruited from 12 sites in Moscow: 2 Tajik diaspora organizations, 4 bazaars, and 6 construction work sites. To be eligible to participate in the research, prospective participants had to be a male Tajik migrant aged 18 or older, a current or former PWID, give written informed consent, intending to reside in Moscow for the next 12 months to permit completing baseline and four follow-up interviews, and willing to recruit two male Tajik migrants who inject drugs for interviewing as PWID network members. Network members (n=280) had to meet the same eligibility criteria as the migrant who referred them but also: 1) have injected drugs at least once in the last 30 days; and 2) be someone whom the referring migrant sees at least once a week to facilitate sharing MASLIHAT HIV prevention information. The analysis draws on data collected from the study’s sample of 420 Tajik migrant male PWID in Moscow at enrollment (baseline) prior to their random assignment to one of two research intervention conditions.

### Data Collection:

After giving informed consent, participants were interviewed at the PRISMA office in Moscow or a private location of their choosing. A structured questionnaire collected information on participants’ sociodemographic characteristics, HIV-related knowledge, HIV risk perception, substance use and sexual risk behavior, experience with police-enacted societal stigma, and psychosocial measures of depression and loneliness. Participants received the customary compensation in Moscow of $20.00 for their time and transportation costs in being interviewed.

### Measures

**Demographic information** consisted of age (years), marital status (currently married, not married/divorced), and highest educational attainment (primary school/secondary school, some university education/higher).

**HIV knowledge** was assessed by responses to 8 statements as being either “safe” or “unsafe” in the possibility of HIV transmission such as “being bitten by mosquitoes or other insects” and 5 statements as either “true” or “false” in terms of becoming infected such as “there is a test to determine if a person has HIV.” Responses were coded as either correct or incorrect with “don’t know” coded as incorrect. Correctly answered items were summed for a possible final score per individual between 0 – 13 (Cronbach alpha = 0.91). Low HIV knowledge was defined as scoring from 0 – 6 and moderate/high as scoring from 7–13.

**Self-perception of HIV risk** was assessed with two items: 1) “How likely are you to get HIV?” on a scale from “not at all likely” (0) to “very likely” (3). 2) How much do you worry about HIV?” from “not at all” (0) to a lot (2). A self-perceived HIV risk score was created per person by summing the responses to both items with scores ranging from 0 – 5. Low HIV risk perception was defined as a score of 0 or 1 and moderate/high as scoring between 2–5.

**HIV sexual risk behavior** was measured with three items: number of female sex partners, sex with a female sex worker, and engaging in condomless sex. To assess possible HIV risk behavior through sexual partnering, participants were asked how many women and the number of female sex workers (FSWs) with whom they had sexual intercourse in the past 30 days. Number of female sex partners was coded as: 0 or 1 sex partner = 0 (little to no risk) or ≥2 sex partners = 1 (some level of risk). Sex with FSW was coded as: 0 FSWs = 0 (no sexual intercourse reported with FSWs) or ≥1 FSWs = 1 (Sex with FSWs). To assess frequency of engaging in sex without a condom: participants were asked, “how often did you use a condom when having sexual intercourse?” for each of three partner categories: “regular female partner in Russia,” “Moscow FSW,” and “other sexual partners not engaged in selling sex.” Response categories were “never,” “sometimes,” “often,” or “always.” Responses of “always used a condom” for all three partner categories were categorized as “engaging in sex with condoms.” Otherwise, responses were categorized as engaging in condomless sex. The items were combined to create a binary measure of “any condomless sex” vs. “sex with condoms” in the past 30 days.

**Psychosocial measures: Symptoms of Depression** were measured using the 20-item Center for Epidemiologic Studies Depression scale - revised (CESD-R).^15,16^ **Loneliness** was measured using the 20-item UCLA loneliness scale and a loneliness score was calculated as the mean of item responses (coefficient alpha: .89 - .94).^17^

**Societal stigma as manifested through police harassment** was measured by responses ranging from “never” (0) to “very often” (4) to each of three statements: “I have been hassled by the police because I’m a migrant,” “I have been detained by the police because I’m a migrant,” and “I have been beaten by the police because I’m a migrant.” A summation score of experience with police-enacted stigma was calculated per participant ranging from 0–12.

### Analysis:

A population-averaged Poisson regression analysis was conducted with a sandwich estimator of variance and exchangeable within-group correlation structure for network clusters to obtain adjusted prevalence ratios (aPRs) with their 95% confidence intervals (95% CI).^17, 18^ Adjusting for demographic and psychosocial factors that might impact HIV sexual risk behavior, multivariable modeling was used to examine associations between HIV knowledge and HIV risk perception and four sexual risk outcomes: sex with multiple partners (model 1), sexual activity with one or more FSWs (model 2), condomless sex (model 3) and condomless sex with FSWs (Model 4). To test the possible moderating effect of HIV risk perception on the relationship between HIV knowledge and sexual risk behavior, the analysis tested for both the separate and interactive effects of HIV knowledge and perception of HIV risk on each sexual outcome. The analyses were performed using STATA 16.1 (Stata, College Station, TX, USA) software.

## Results

Table 1 presents both the demographic characteristics of the total sample and the subsample of participants reporting sexual activity in the 30 days prior to being interviewed. The total sample was on average 30 years of age, not married/divorced, and at a secondary school education level. No significant differences were found between the demographic characteristics of men recruited through worksites, bazaars, and non-governmental organizations (NGOs) versus those whom they referred to the study as network members with the exception that the latter on average were one year younger (B = −1.07, 95% CI −1.69 – −0.46) and more likely to be on their first labor migration trip (Wald χ^2^ = 7.49, p = .02).

### Sexual Risk Behavior.

Of the total 420 participants in the study sample, 60.7% (255) reported having been sexually active in the last 30 days prior to being interviewed. Of these, 124 men (48.6%) reported having sex with multiple partners, 177 (69.4%) reported sex with one or more FSWs, and 178 (69.8%) reported engaging in sex without a condom including 62 men (35%) who did not use one when having sex with an FSW.

### HIV Knowledge.

Of the 420 participants in the study, 136 (32.4%) scored low on HIV knowledge including 88 (34.5%) of the 255 men who reported sexual activity in the last 30 days. Of the 88 sexually active men who also scored low on HIV knowledge, 45 (51.1%) reported having multiple partners, 62 (70.4%) reported sex with a FSW, and 61 (69.3%) reported engaging in condomless intercourse including 22 participants who reported not having used one with FSWs.

### Self-perception of HIV Risk.

Of the 420 participants in the study, 189 (45.0%) perceived themselves to be at low risk for HIV. Of the 123 sexually active men who also perceived themselves to be at low HIV risk, 82 (66.7%) reported having multiple sex partners, 97 (78.9%) reported sexual activity with FSWs, and 83 (67.5%) reported engaging in condomless sex including 39 men who reported sexual activity with an FSW.

### Multivariate analyses:

All study participants for whom there was complete data were included in the multivariable modeling of factors associated with engaging in sexual risk. Table 2 shows the adjusted prevalence ratios of each sexual risk behavior. Model 1 and 2 examine the association between key variables and engaging in sex with multiple sex partners and with FSWs. Low HIV risk perception was associated with a 79% higher prevalence of multiple sex partners (aPR: 1.79, 95% CI: 1.33, 2.40, Table 2) and a 28% higher prevalence of sexual activity with FSWs (aPR: 1.28, 95% CI: 1.04, 1.59) when compared to those with moderate/high HIV risk perception. Older age was associated with a lower prevalence of multiple sex partners (aPR: 0.87, 95% CI: 0.84, 0.91) and sexual activity with FSWs (0.91, 95% CI: 0.88, 0.94). A one-unit increase in depression scoring was associated with a 19% lower prevalence of multiple sex partners (aPR: 0.81, 95% CI: 0.65, 1.01). Conversely, a one-unit increase in police-enacted stigma score was associated with a 22% greater prevalence of multiple sex partners (aPR: 1.22, 95% CI: 1.01, 1.49) and a 20% greater prevalence of sexual activity with FSWs (aPR: 1.20, 95% CI: 1.03, 1.40).

Model 3 and Model 4 investigate condomless sex generally as well as specifically with FSWs. Neither knowledge of how HIV is transmitted, nor HIV risk perception were significantly associated with condomless sex or condomless sex with FSWs. Meanwhile, higher levels of loneliness were associated with less likelihood of engaging in condomless sex (aPR: 0.79, 95% CI: 0.68, 0.92), while a one-unit increase in depression score was associated with a 14% higher likelihood of engaging in condomless sex (aPR: 1.14, 95% CI: 1.05, 1.24) and a 26% higher likelihood of engaging in condomless sex with FSWs specifically (aPR: 1.26, 95% CI: 1.03, 1.54).

The joint effect of HIV knowledge and HIV risk perception on each sexual risk outcome is shown in Table 3. No evidence of an additive interaction between HIV knowledge and HIV risk was found. When low HIV risk perception was present, however, the prevalence of multiple sex partners, sexual activity with FSWs, and condomless sex with FSWs was higher.

## Discussion

To the best of our knowledge, this is the largest cross-sectional study to examine the factors that may influence HIV sexual risk behavior among male Tajik MWIDs as a highly understudied population. Prevalence of HIV sexual risk behavior among this migrant population is high as evidenced by the number of men engaging in sex with multiple partners (48.6%) and sex without a condom (69.8%) including with FSWs (35.0%). Of the 420 men participating in the study, 136 (32.4%) scored low on HIV knowledge and 189 (46.1%) on self-perceived HIV risk.

Prevention programs for at-risk populations typically build on the assumption that knowledge of which sexual activities carry HIV risk is needed to reduce or end high risk behavior. Yet, the study’s finding that HIV knowledge was not associated with engaging in risky sexual behavior suggests that providing Tajik migrants with HIV risk-reduction education alone likely would prove ineffective. Meanwhile, low self-perceived risk for HIV was found to be associated with greater likelihood of engaging in risky sexual activity as indicated by multiple partners and condomless sex including with FSWs. Analysis of the possible joint influence of HIV knowledge and self-perceived risk for HIV on risky sexual behavior failed to support an interaction effect. These findings suggest the role of self-perceived HIV risk over HIV knowledge as a critical factor influencing sexual risk behavior. HIV prevention programming designed to raise Tajik migrants’ and possibly other vulnerable populations’ awareness of being at personal risk is needed to strengthen the effects of HIV preventive interventions.

As for psychosocial-related factors, depression and police-enacted stigma were positively associated with sexual activity with multiple sex partners and FSWs in the last 30 days, whereas loneliness and depression were associated with condomless sex. Yet, the prevalence of all four psychosocial variables as reported in Table 1 was marginally higher among men who disclosed not having engaged in sex versus those who were sexually active during the same 30-day period. The similarity between the two sub-populations suggests that Tajik migrants in general are subject to emotional distress that can negatively affect their daily lives. Possibly among migrant males who are sexually active, these negative feelings can manifest themselves in the increased likelihood that some men will engage in less than safe sexual behavior. Whatever the reason, however, the correlation of these psychosocial with risky sex calls attention to the need for HIV prevention programming that helps to address the stress and psychosocial challenges of migrant life.

### Limitations

The study’s analysis of the prevalence and correlates of risky sex focuses solely on those who reported sexual activity and excludes those men who reported being sexually inactive. It may be that some unknown number of the study’s sexually inactive participants purposely abstained from sex to avoid HIV, but the study’s data do not include inquiry into this possibility. Also, neither of the two measures used in asking participants to assess their personal risk for HIV specified that this calculation should be based solely on sexual risk. Given that the study’s sample is composed entirely of PWID, some proportion of participants may have factored in personal risk for HIV through both sex and injection drug use. While this likelihood doesn’t mitigate the validity of the study’s findings derived by examining the association between self-perceived risk for HIV and engaging in risky sexual behavior, it does not allow assessment of how much drug use alone may contribute to Tajik MWID’s self-perception of being at HIV risk.

In terms of sampling, the study’s initial participants were obtained through in-person recruitment at 10 occupational sites and by referral from 2 Tajik service networks. Each of these participants, in turn, recruited two active MWID participants through their social networks. Consequently, the results of the study may not generalize to those migrants whom these methods failed to reach or who chose not to participate. Also, our sample consists solely of men, so we cannot comment on sexual risk or its correlates among the small but growing number of Tajik women who migrate to Moscow for work. In addition, the study’s measures of sexual risk behavior focus solely on unsafe sex with female sexual partners. It is quite possible that acts of unsafe sex with another male also may have occurred during the same 30-day period. Same-sex behavior is highly stigmatized and considered morally unacceptable within the male Tajik migrant community, and it is unlikely that its occurrence would be disclosed by a participant during an initial interview. Moreover, asking a Tajik male about possibly having engaged in same-sex behavior is sufficiently affrontive culturally that it carries the potential risk of triggering the participant’s decision to end the interview. Consequently, measures of sexual risk in the study are confined solely to unsafe sex with female partners. Finally, the study’s baseline data are cross-sectional and only examined HIV risk behavior in the last 30 days. They cannot provide information about how variables of interest may have differed prior to or after this measurement period.

## Conclusions

The study’s results underline the need for culturally relevant, prevention programing and other relevant interventions for male Tajik MWIDs that go beyond increasing HIV knowledge to also promote evidence-based awareness of personal risk for HIV. Additionally, psychological services to counter the psychosocial effects of loneliness, depression, and societal stigma including through police harassment are needed.

## Figures and Tables

**Table 1.** Demographic Characteristics & HIV Sexual Risk Behavior of Male Tajik MWID

Study Variable^1^	Total Population, N=420			Among Those Reporting Sexual Activity, N=255		
	*Range*	*Mean*	*SD*	*Range*	*Mean*	*SD*
*Main Study Factors*						
HIV Knowledge Score	0–13	7.21	2.30	0–13	7.05	3.32
HIV Risk Perception Score	0–5	1.75	1.35	0–5	1.61	1.17
		***n***	***%***		***n***	***%***
Low HIV Knowledge	--	136	32.4	--	88	34.5
Low HIV Risk Perception	--	189	45.0	--	123	49.2
*Psychosocial Factors*						
Loneliness	19–68	43.54	12.33	19–68	41.23	12.14
Depression	0–4	0.90	0.97	0–4	0.76	0.83
Police-Enacted Stigma	0–8	2.70	2.05	0–8	2.58	2.06
*Demographic Factors*						
Age	19–50	29.90	6.20	19–50	27.98	5.61
		***n***	***%***		***n***	***%***
Married	--	52	12.4	--	19	7.5
Highest Education						
Primary	--	16	3.8	--	7	2.8
Secondary	--	240	57.1	--	177	69.4
College or Technical	--	105	25.0	--	54	21.2
University (No degree)	--	14	3.3	--	6	2.4
University Degree	--	45	10.7	--	11	4.3
*Main Study Outcomes*						
		***n***	***%***		***n***	***%***
Multiple Sex Partners^2^	--	125	29.8	--	124	48.6
Sex with FSW	--	177	42.1	--	177	69.4
Condomless Sex	--	178	42.4	--	178	69.8
Condomless Sex with	--	62	14.8	--		35.0
FSW					62^3^	

1 Frequency of study characteristics may not sum to column N due to missingness

2 One individual reporting multiple sex partners did not complete information to assess condomless sex

3 Among those reporting sex with FSW, N=177

**Table 2.** Adjusted Associations with Number of Sex Partners and Condomless Sex Among Male Tajik MWID

Study Variable	Model 1: Multiple Sex Partners, N=410	Model 2: Sex with FSW, N=410	Model 3: Condomless Sex, N=250	Model 4: Condomless Sex with FSW, N=172
	*aPR (95% CI)*	*aPR (95% CI)*	*aPR (95% CI)*	*aPR (95% CI)*
Low HIV Knowledge	0.92 (0.69, 1.21)	0.98 (0.79, 1.20)	1.03 (0.87, 1.21)	0.92 (0.63, 1.34)
Low HIV Risk Perception	1.79 (1.33, 2.40)	1.28 (1.04, 1.59)	0.99 (0.84, 1.16)	1.49 (0.88, 2.52)
Age	0.87 (0.84, 0.91)	0.91 (0.88, 0.94)	1.02 (1.01, 1.03)	1.04 (1.01, 1.09)
Loneliness	1.06 (0.87, 1.31)	1.18 (0.96, 1.44)	0.79 (0.68, 0.92)	0.82 (0.52, 1.31)
Depression	0.81 (0.65, 1.01)	0.88 (0.75, 1.02)	1.14 (1.05, 1.24)	1.26 (1.03, 1.54)
Police-Enacted Stigma	1.22 (1.01, 1.49)	1.20 (1.03, 1.40)	0.91 (0.80, 1.04)	1.28 (0.92, 1.79)
Married	0.38 (0.14, 0.99)	0.70 (0.45, 1.08)	0.74 (0.48, 1.15)	0.33 (0.04, 2.55)
Highest Education				
Primary/Secondary	1.00	1.00	1.00	1.00
Some College/Above	0.78 (0.58, 1.04)	0.74 (0.58, 0.94)	0.84 (0.67, 1.05)	0.79 (0.48, 1.28)

1 Wald χ^2^

**Table 3.** Joint Effect of HIV Knowledge and HIV Risk Perception Among Male Tajik MWID

HIV Knowledge	HIV Risk Perception	N (%)	% Reporting Outcome	Prevalence Ratio aPR (95% CI) `	RERI (95% CI) ^2^
*Model 1: Multiple Sex Partners, N=410*					
Moderate/High	Moderate/High	26 (16.4)	16.4	ref	-
Low	Moderate/High	14 (22.6)	22.6	0.94 (0.34, 1.45)	--
Moderate/High	Low	52 (44.1)	44.1	1.82 (1.21, 2.76)	--
Low	Low	30 (42.3)	42.3	1.65 (1.01, 2.74)	−0.11 (−0.70, 0.60)
*Model 2: Sex with FSW, N=410*					
Moderate/High	Moderate/High	52 (32.7)	32.7	ref	--
Low	Moderate/High	23 (37.1)	37.1	0.89 (0.55, 1.28)	--
Moderate/High	Low	59 (50.0)	50.0	1.19 (0.87, 1.49)	--
Low	Low	38 (53.5)	53.5	1.21 (0.89, 1.70)	0.13 (−0.27, 0.64)
*Model 3: Condomless Sex, N=250*					
Moderate/High	Moderate/High	61 (70.9)	70.9	ref	--
Low	Moderate/High	31 (75.6)	75.6	1.06 (0.88, 1.26)	--
Moderate/High	Low	53 (68.8)	68.8	1.01 (0.90, 1.30)	--
Low	Low	30 (65.2)	65.2	0.96 (0.72, 1.24)	−0.10 (−0.58, 0.15)
*Model 4: Condomless Sex with FSW, N=172*					
Moderate/High	Moderate/High	14 (26.9)	26.9	ref	--
Low	Moderate/High	7 (30.4)	30.4	1.14 (0.40, 2.29)	--
Moderate/High	Low	24 (40.7)	40.7	1.68 (0.80, 3.61)	--
Low	Low	15 (39.5)	39.5	1.63 (0.79, 2.93)	−0.19 (−2.22, 1.05)

` All estimates adjusted for study variables in modified Poisson regression

## Data Availability

The data will be made publicly available upon completion of the study. Information on how to access the data may be obtained by contacting the corresponding author.

## Abbreviations

HIV: human immunodeficiency virus
MWID: migrants who inject drugs
PWID: people who inject drugs
AIDS: acquired immunodeficiency syndrome
FSW: female sex workers
CESD-R: Center for Epidemiologic Studies Depression scale – revised
aPR: adjusted prevalence ratios
95% CI: 95% confidence intervals
NGO: Non-governmental organization
